# Cancer genomics: one cell at a time

**DOI:** 10.1186/s13059-014-0452-9

**Published:** 2014-08-30

**Authors:** Nicholas E Navin

**Affiliations:** Department of Genetics Unit 1010, The University of Texas MD Anderson Cancer Center, 1515 Holcombe Blvd, Houston, TX 77030 USA; Department of Bioinformatics and Computational Biology, The University of Texas MD Anderson Cancer Center, 1515 Holcombe Blvd, Houston, TX 77030 USA; The University of Texas Graduate School of Biomedical Sciences, 1515 Holcombe Blvd, Houston, TX 77030 USA

## Abstract

The study of single cancer cells has transformed from qualitative microscopic images to quantitative genomic datasets. This paradigm shift has been fueled by the development of single-cell sequencing technologies, which provide a powerful new approach to study complex biological processes in human cancers.

## Introduction

Biologists have been studying single cancer cells since the invention of the microscope by Antonie van Leeuwenhoek in 1665. Many initial observations were based on the morphological differences between tumor cells, as recorded in the late 1800s by early pathologists, such as Rudolf Virchow [1]. These observations were greatly improved by the development of cellular staining techniques, such as hematoxylin and eosin. In the 1980s, the development of cytogenetic techniques, including spectral karyotyping (SKY) and fluorescence *in situ* hybridization (FISH), galvanized the field by allowing researchers to visualize the genomic diversity of chromosome aberrations directly in single tumor cells [2–4]. However, only in the past four years has the field moved from qualitative imaging data to quantitative datasets that are amenable to statistical and computational analysis. This paradigm shift has largely been fueled by the development of whole-genome amplification (WGA) and whole-transcriptome amplification (WTA), methods that can amplify the genome or transcriptome of a single cell from picogram-to-microgram quantities. By combining these methods with next-generation sequencing (NGS) technologies, it is now possible to obtain genome-wide mutational and transcriptional datasets on individual cancer cells.

Single-cell sequencing (SCS) promises to address key issues in cancer research, including resolving intratumor heterogeneity, tracing cell lineages, understanding rare tumor cell populations and measuring mutation rates. Such investigations were previously difficult to perform by sequencing bulk tissue samples, as these are limited to providing an average signal from a complex population of cells. While some clonal diversity can be resolved by deconvoluting deep-sequencing data [5–7] and sequencing different spatial regions of tumors [8], the data still reflect an admixture signal. The presence of multiple clonal subpopulations and rare tumor cells is difficult to resolve from these data, and determination of which combinations of mutations are present in any given cell is also hard to resolve. In addition to the genomic heterogeneity within tumors, there is also phenotypic heterogeneity, which can be caused by genomic mutations, or through epigenetic modifications, transcriptional changes, alterations in protein levels or protein modifications. Most notably, many solid tumors show evidence of harboring both epithelial and mesenchymal populations, the latter of which are often referred to as cancer stem cells. These stem-like cells are clear progenitors in hematopoietic cancers, but remain a controversial subject with respect to most solid tumors [9–11].

**Figure 1:**
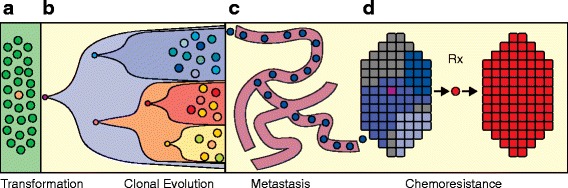
**Single-cell processes in cancer.** Although single cancer cells interact with their neighbors and the adjacent stromal cells, there are many biological processes that occur through the actions of individual cancer cells, shown in this illustration. These complex biological processes in human cancers include: **(a)** transformation from a single normal somatic cell into a tumor cell; **(b)** clonal evolution that occurs through a series of selective sweeps when single cells acquire driver mutations and diversify, leading to intratumor heterogeneity; **(c)** single cells from the primary tumor intravasate into the circulatory system and extravasate at distant organ sites to form metastatic tumors; and **(d)** the evolution of chemoresistance that occurs when the tumor is eradicated but survived by single tumor cells that harbor resistance mutations and expand to reconstitute the tumor mass.

While there is substantial evidence that tumor cells can communicate with their neighbors and the stroma, there are also many complex biological processes that occur through the actions of individual cancer cells. These processes include the initial transformation event in a normal cell, clonal expansion within the primary tumor, metastatic dissemination and the evolution of chemoresistance (Figure 1). SCS provides a powerful new approach to study the genomic and transcriptomic basis of these processes directly in human cancers, without the necessity for model organisms.

In this review, we discuss how SCS approaches are helping to resolve fundamental questions in cancer biology, including: what is the range and extent of clonal diversity in human cancers? Do tumors evolve from single cells in normal tissues, or from multiple cells? Do tumor cells have an increased mutation rate relative to normal cells? Which clones are responsible for metastatic dissemination and evolving resistance to chemotherapy, and are they rare? Several groups have begun to address questions such as these by using SCS in a variety of cancers, but many technical hurdles still remain in order to distinguish real biological diversity from technical errors. We will discuss the advantages and caveats of different SCS techniques, as well as their applications to clinical practice.

## Isolating a single cancer cell

**Figure 2:**
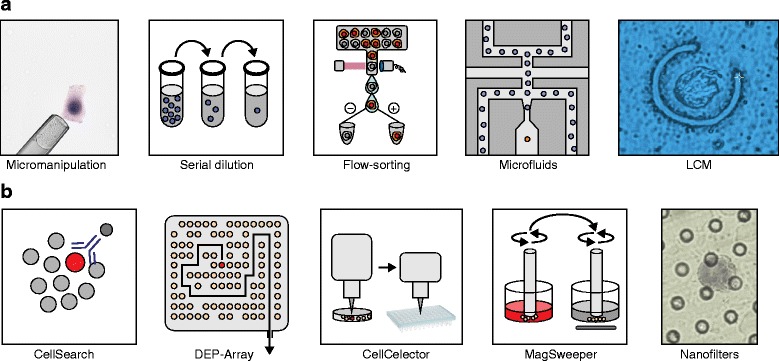
**Methods for isolating single cancer cells from abundant and rare populations. (a)** Methods for isolating single cells from abundant cellular populations include: micromanipulation by robotics or mouth pipetting, serial dilutions, flow-sorting, microfluidics platforms and laser-capture microdissection (LCM; 63X objective). **(b)** Methods for isolating single cells from rare cellular populations include: CellSearch (Johnson & Johnson), DEP-Array (Silicon Biosciences), CellCelector (Automated Lab Solutions), MagSweeper (Illumina) and nano-fabricated filters (Creatv MicroTech).

In order to study a single cancer cell, the cell must first be isolated from the population. Several well-established methods can be used to isolate single cells that are abundant in a population, including micromanipulation, serial dilution, flow-assisted cell sorting (FACS), microfluidic devices and laser-capture microdissection (LCM) (Figure 2). The advantages and caveats of these collection methods have been reviewed previously [9]. It is important to note that most of these methods require suspensions of cells prepared from fresh cancer tissue. It is often not possible to obtain cell suspensions as most archival tumor samples have been flash-frozen or formalin-fixed paraffin-embedded (FFPE). Freezing often leads to rupture of the cytoplasmic membrane, but frequently leaves the nuclei intact. To circumvent these problems, several studies [10–12] have shown that single nuclei can be isolated for SCS applications, often referred to as single-nucleus sequencing (SNS). Alternatively, LCM methods can preserve the spatial location of cancer cells in the context of their tissue geography. However, LCM introduces a number of technical artifacts, including slicing the cells during the preparation of tissue sections and UV damage to DNA or RNA from the laser cutting energy [13].

While the aforementioned methods are efficient at isolating single cells from an abundant population, the isolation of rare cancer cells (<1% of the total tumor cell population) remains difficult. This is particularly problematic as there is great interest in the field in isolating circulating tumor cells (CTCs), disseminated tumor cells (DTCs) and cancer stem cells (CSCs) in order to understand their role in tumor progression and metastasis. CTCs and DTCs can occur at very low frequencies (one in one million mononuclear cells) in the blood or bone marrow [12,13].

Several new technologies have been developed to isolate rare CTCs or DTCs from the blood using fluorescent markers. The CellSearch magnetic bead system (Johnson & Johnson) was the first clinical system developed to detect and enumerate CTCs in blood samples and is widely used in the clinic today [14]. This system uses magnets with ferrofluid nanoparticles conjugated to the antibodies EpCAM and CD45 to enumerate or isolate CTCs. EpCAM is an epithelial marker that is present on epithelial tumor cells, but absent in most blood cells. CD45 is an immunocyte marker that is present on many blood cells, but absent in the CTCs. The DEP-Array system (Silicon Biosciences) uses a microfluidics chip with dielectropheretic cages to navigate individual cells by charge after identification with fluorescent markers [15]. The advantage of this system is that every cell is preserved, and even a single cell in a pool of 100,000 can be isolated efficiently. Another method, called the CellCelector (Automated Lab Solutions), uses nanofabricated wells to isolate and phenotype single cells that can then be isolated by a robotic micromanipulator [16]. This system is high-throughput but requires that single cells be diluted in suspensions for capture. The nanopost microchip technology involves flowing CTCs through a series of posts to which antibodies against EpCAM have been conjugated [17]. Another technology, called Magsweeper (Illumina), involves dipping a rotating magnet with bound EpCAM antibodies in order to isolate CTCs and then moving the magnet into a new buffer for release of the CTCs [18]. The caveat of the aforementioned methods is that they depend on identifying rare cells using fluorescent markers, and thus are highly biased. In CTCs, cells are generally selected as EpCAM-positive and CD45-negative, which would miss any tumor cells with a mesenchymal phenotype. An alternative method, which overcomes this problem, involves isolating rare tumor cells by size discrimination on nanofabricated filters (CellSieve) [19]. The principle underlying this method is the fact that most CTCs are larger in size (>7 μm) than the white blood cells (<7 μm) and thus can be filtered by size discrimination. In summary, none of the technologies discussed is perfect for isolating rare tumor cells, and careful considerations must be taken in order to avoid biasing the population of single cells that are selected or missing them entirely.

## Single-cell sequencing technologies

SCS technologies have evolved substantially in the area of genome and transcriptome sequencing over the past four years, a technical feat that was considered inconceivable only a few years ago. The development of single-cell RNA-seq methods has shown significant progress owing to the fact the each single cell harbors thousands of copies of each mRNA transcript, while having only two copies of each chromosomal DNA molecule. Consequently, the field has seen a proliferation of methods for performing single-cell RNA-seq [20–25], overcoming many of the initial technical challenges, including amplification distortions, obtaining full-length transcripts and mitigating 3′ bias. Single-cell RNA sequencing methods (summarized in Table 1) have been reviewed in detail elsewhere [26,27].

**Table 1:** **Single-cell sequencing methods** ^**a**^

**Approach** ^**b**^	**WGA method** ^**c**^	**Enzyme** ^**d**^	**Cells/nuclei** ^**e**^	**Applications** ^**f**^	**Coverage breadth** ^**g**^	**Commercial kits** ^**h**^	**References**
**SCS DNA-seq**							
SNS	DOP-PCR	Thermosequenase	Nuclei	Copy number profiling	~10%	WGA4 Sigma	[34]
MALBAC	DOP-PCR	Bst	Cells	Copy number profiling	>90%	Bst NEB	[31]
BGI MDA	MDA	Phi29	Cells	Genome/exome	>90%	Repli-G Qiagen	[30]
NUC-SEQ	MDA	Phi29	Nuclei	Genome/exome	>90%	Repli-G Qiagen	[37]
**SCS RNA-seq**							
Tang method	PolyA priming	Reverse transcriptase	Cells	Transcriptome	3' bias	NA	[21]
Quartz-seq	PolyA priming	Reverse transcriptase	Cells	Transcriptome	3' bias	NA	[24]
CEL-seq	PolyA priming	Transcription *in vitro*	Cells	Transcriptome	3' bias	NA	[20]
STRT-seq	Template-switching	Reverse transcriptase	Cells	Transcriptome	Full-length	NA	[23]
Smart-seq	Template-switching	RT MMLV	Cells	Transcriptome	Full-length	Clontech	[22]
PMA	MDA	Phi29	Cells	Transcriptome	3' bias	NA	[25]

^a^Table summarizes the methods for single-cell DNA sequencing and single-cell RNA sequencing. ^b^Name of the method; ^c^amplification method; ^d^enzyme used for amplification; ^e^description of whether the method was designed for analysis of cells or nuclei; ^f^description of the type of molecular information that is best measured using the method; ^g^reference to the total number of bases that can be covered with sequencing data using the approach; ^h^indication of whether any commercially available kits have been developed to perform the method. *Abbreviations*: *BGI* Beijing Genome Institute, *Bst* *Bacillus stearothermophilus* DNA polymerase, *DOP-PCR* degenerative-oligonucleotide PCR, *MALBAC* multiple annealing and looping-based amplification cycles, *MDA* multiple-displacement amplification, *NA* not applicable, *PCR* polymerase chain reaction, *PMA* Phi29 DNA-polymerase-based mRNA transcriptome amplification, *RT MMLV* reverse transcriptase Moloney murine leukemia virus, *SNS* single-nucleus sequencing, *WGA* whole-genome amplification.

**Figure 3:**
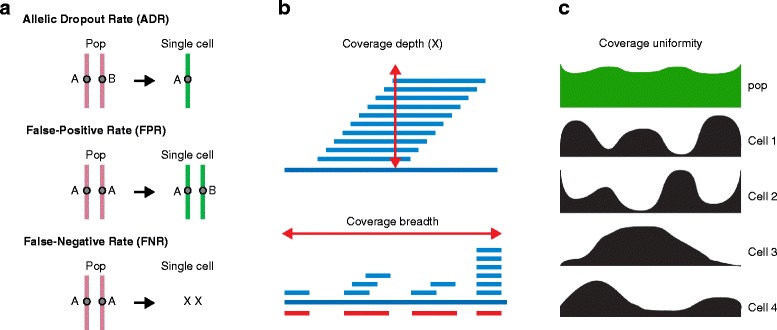
**Technical errors and coverage in single-cell sequencing data. (a)** Technical errors that occur in single-cell sequencing (SCS) data include: false-positive errors, allelic dropout events and false-negative errors due to insufficient coverage. ‘Pop’ indicates a population of cells. **(b)** Coverage metrics in SCS data include coverage depth and total physical coverage, or breadth. **(c)** Coverage uniformity, or ‘eveness’ in SCS data can vary from cell to cell, but is often more uniform in standard genomic DNA sequencing experiments using populations of cells.

By contrast, the development of single-cell genome and exome sequencing methods has proved to be more challenging and will be discussed in detail. Starting with only two copies of DNA as input material for WGA results in a number of technical errors, including low physical coverage, non-uniform coverage, allelic dropout (ADO) events, false-positive (FP) errors and false-negative (FN) errors due to insufficient coverage (Figure 3). In sequencing the genome or exome of a single cell, it is often difficult to achieve high coverage breadth (nucleotide sites with at least 1X coverage). However, achieving high physical coverage of the exons or genome is crucial for calling mutations at the same regions across multiple single cells. Coverage uniformity (or ‘evenness’) is another technical challenge with single-cell data, owing to the significant GC bias that occurs during WGA (Figure 3c). This leads to deviations from the Poisson coverage distributions that are normally observed in NGS data, requiring higher coverage depths to achieve sufficient coverage in regions with low read counts. FP errors occur due to the infidelity of the WGA polymerase during amplification and lead to single-base-pair errors [28,29] (Figure 3a). These errors are most severe during the initial rounds of genome duplication because all subsequent molecules inherit the errors, making them abundant in the pool. Interestingly, most FP errors generated during a WGA approach called multiple-displacement amplification (MDA) show a very strong bias for C > T (G > A) transitions [30], which could be mitigated by filtering or using probabilistic variant calling models. However, by far the greatest errors that plague SCS data are ADO events, which can be found in 10 to 50% of the mutation sites [28,30–33]. ADO occurs when one allele in a heterozygous mutation (AB) is not amplified by the polymerase, resulting in a homozygous genotype (AA or BB) (Figure 3a). These technical errors must be accounted for in post-processing analysis of SCS data as otherwise every mutation will be reported as showing heterogeneity in the population of single cells.

Importantly, WGA is not a single technique, but encompasses a wide variety of experimental methods and polymerases (Table 1). The most common WGA methods used in SCS studies include degenerative-oligonucleotide-PCR (DOP-PCR) and MDA using either the Phi29 or Bst bacteriophage polymerases. DOP-PCR generates low physical coverage of a single-cell genome (approximately 10%) but can accurately retain copy number levels during amplification, which makes it an ideal method for single-cell copy-number profiling. This approach was used in the first SCS method developed, called single-nucleus sequencing (SNS), to generate high-resolution (54 kb) copy-number profiles from sparse NGS data [34,35]. However, the low physical coverage of DOP-PCR in a single cell makes it a poor tool for measuring mutations at base-pair resolution. MDA using either the Phi29 or Bst polymerases can achieve high-coverage (>90%) sequencing data from the genome or exome of a single cell [30,31,36,37]. However, the caveat of MDA is that it generates non-uniform coverage and can therefore result in very high distortions of copy-number states. Phi29 is the ideal polymerase for MDA reactions as it has an error rate of 10^-7^, whereas Bst has a much higher per base error rate at 10^-5^ [29,38]. Technical errors accumulate during the WGA reaction, resulting in hundreds of thousands of FP errors in the genome of each single cell. SCS methods using the Phi29 polymerases have estimated that the final FP error frequency (approximately 2.5 × 10^-5^) would approximate to >160,000 technical errors in each human single-cell genome [25,26]. Many FP errors occur randomly and can be mitigated by calling mutations that occur in two or more cells at the same nucleotide site; however, recurrent errors cannot be eliminated with this approach.

Another SCS DNA method that has been developed is called ‘multiple annealing and looping-based amplification cycles’ (MALBAC) and uses the Bst polymerase to form circular DNA fragments followed by adapter ligation PCR (Table 1). While the idea of forming circular DNA molecules to inhibit further amplification is elegant, the initial study did not provide experimental evidence supporting this phenomenon [31]. If circular DNAs were in fact formed and did not serve as further templates, the method would be expected to generate extremely low FP error rates as each newly synthesized molecule would contain random errors that are not propagated. However, MALBAC holds the highest FP error rate of all of the SCS methods, probably due to the high infidelity of the Bst polymerase (10^-5^) [31]. For this reason, MALBAC is more useful for copy-number profiling applications than for the detection of point mutations or indels at base-pair resolution (similar to other DOP-PCR-based methods such as SNS). Another method, called NUC-SEQ, uses cells in G2/M phase of the cell cycle to duplicate the amount of starting material in a single cell from 6 pg to 12 pg, followed by limited isothermal amplification using the Phi29 polymerase and tagmentation to generate libraries for NGS [29]. This approach improves physical coverage (>94%) and reduces the ADO (approximately 10%) and FP error rate of SCS by limiting the isothermal amplification timeframe for WGA [37] (Table 1).

In summary, the DOP-PCR-based WGA methods and MALBAC are ideal for copy-number profiling as they generate very high FP error rates and low physical coverage, but provide uniform amplification across the genome. In contrast, the Phi29-based MDA methods are more suitable for the detection of point mutations and indels at base-pair resolution. However, owing to the high technical error rates, mutations must be detected in multiple single cells in order to distinguish real biological variants from technical errors. Furthermore, validation of individual mutations or transcriptional changes using an orthogonal technology is imperative at this stage of the sequencing technologies. An excellent review on the technical details of WGA and WTA methods has been published elsewhere [39].

## Intratumor heterogeneity and clonal evolution in primary tumors

Intratumor heterogeneity has been widely reported in many human cancer types [7,8,30] and confounds the clinical diagnosis and therapeutic targeting of tumors. Intratumor heterogeneity is generally viewed as ‘bad news’ from a clinical standpoint because single samples might not represent the tumor as a whole. However, the genomic diversity within tumors provides an excellent opportunity to study genome evolution because it provides a permanent record of the mutations that occurred during the natural history of the tumor. By assuming that mutational complexity increases with time, we can apply phylogenetic methods to reconstruct the relative chronology of mutations [40]. The first study to use this approach involved applying SNS to study the evolution of aneuploidy in patients with triple-negative (ER^-^/PR^-^/HER2^-^) breast cancers (TNBCs; negative for, respectively, the estrogen receptor, progesterone receptor and the receptor tyrosine-protein kinase erbB-2 (HER2)) [34]. This involved undertaking a comparative analysis of 100 single-cell copy-number profiles from two patients with TNBC, which revealed that copy-number aberrations (CNAs) evolved in punctuated bursts of evolution, followed by stable clonal expansions to form the tumor mass. These data challenged the prevailing model that mutations accumulate gradually and sequentially over extended periods of time, leading to more-malignant stages of cancer [41]. Also identified were four rare tumor cells that showed a 50-fold amplification of the *KRAS* (Kirsten rat sarcoma viral oncogene homolog) locus that was absent in the major tumor subpopulations, suggesting that the most malignant populations in the tumor might also be the rarest.

Although SNS is adequate for copy-number profiling, it cannot accurately resolve mutations at base-pair resolution owing to low physical coverage (approximately 6%) in each single-cell genome. To address this problem, an MDA-based method was developed called NUC-SEQ that can be used to perform high-coverage, whole-genome and exome sequencing of individual nuclei [37]. NUC-SEQ was applied to study copy-number and mutational evolution in two breast cancer patients: a TNBC patient and an ER-positive breast cancer patient. In both tumors, the data suggested that copy-number rearrangements evolved early, in punctuated bursts of evolution, followed by stable expansions to form the tumor masses. By contrast, point mutations evolved gradually over extended periods of time, generating extensive clonal diversity. The single-cell exome sequencing data also identified many rare subclonal mutations that were validated by targeted deep sequencing (>140,000X) using a molecular barcoding approach called duplex sequencing [42] to decrease the error rate of NGS from 10^-2^ to 10^-10^. The data suggested that many subclonal mutations were present at low mutation frequencies (<1%) in the tumor mass, possibly diversifying the phenotypes of cancer cells. These rare subclonal mutations might be important when the tumor cells encounter selective pressures in their microenvironment, such as the immune system, hypoxia, nutrient deprivation or chemotherapy [43,44].

Single-cell exome sequencing has also been used to study clonal diversity and tumor evolution in several other human cancer types. Two controversial studies from the Beijing Genome Institute (BGI) involved sequencing a renal carcinoma [36] and a myeloproliferative neoplasm [30]. The authors performed exome sequencing of 25 single cells from the renal cell carcinoma and compared point mutations between the cells, from which they concluded that no population substructure was evident and indeed the tumor mass consisted of a monoclonal population of cells. Similarly, in the study of *JAK2*-positive myeloproliferative neoplasms, the authors compared exome-wide point mutations of 58 cells and postulated that the tumor evolved from a ‘monoclonal origin’ representing a monoclonal population of tumor cells. The data and conclusions in these studies are contradicted by the phylogenetic trees, which show large genetic distances existing between individual tumor cells. This genetic distance might be due to the high technical error rates of the method or due to real biological cell-to-cell genetic variation, but could not be resolved in these datasets. To deal with the high technical error rates, the authors decided to combine all of the single-cell data and identified mutations that occur in the majority of the tumor cells, which is conceptually equivalent to sequencing the bulk tumor *en masse*.

While the utility of single-cell exome sequencing data in lineage-tracing studies was not established in the original studies, researchers from the BGI have recently applied the same method to sequence 66 single cells from a muscle-invasive bladder cancer, in which two major tumor subpopulations were found to have diverged from a common genetic lineage [45]. This lineage is likely to be accurate as a large number of single cells with distinct sets of mutations were identified from two major subpopulations, and the data show that both subpopulations share a large number of founder mutations, suggesting evolution from a common origin. In another recent BGI study, the authors sequenced 63 single cells from a patient with colon cancer and used hierarchical clustering to show that two groups of tumor cells were present, from which they concluded that the tumor evolved from a ‘biclonal’ origin [46]. A biclonal origin, in the strictest sense of the definition, suggests that a tumor evolved from two independent normal cells in the colon tissue and therefore would not be expected to share any common mutations in their genetic lineages. However, a biclonal model is contradicted in these data by the many single cells from each lineage that share several prominent point mutations (for example, *PABPC1* and *CDC27*) that are highly unlikely to have arisen independently through convergent evolution. In summary, constructing accurate cell lineages from single-cell exome data still remains challenging owing to the high FP and ADO error rates in these studies.

SCS has also shown great value in tracing cell lineage in hematopoietic cancers, including acute myeloid leukemia (AML). In contrast to the aforementioned studies, these studies used targeted sequencing of gene panels in single AML tumor cells, which allows more cells to be profiled and at a lower cost. One study profiled single cells from three patients diagnosed with MDS (myelodysplastic syndrome)-derived secondary AML that were previously analyzed by whole-genome sequencing [47]. The SCS data agreed very well with the clonal-substructure predictions from the deep-sequencing data and, furthermore, showed which combination of mutations was present in each individual cell. This allowed the authors to build phylogenetic trees and reconstruct the order of mutations that occurred as the clones evolved from progenitor subpopulations. In another study using targeted SCS, the authors showed that self-renewing hematopoietic stem cells (HSCs) underwent clonal evolution, accumulating founder mutations in *FLT3-ITD* (receptor-type tyrosine protein kinase internal tandem duplications) followed by sequential mutations in *NPM1* (encoding nucleophosmin), *TET2* (encoding methylcytosine dioxygenase) and *SMC1A* (structural maintenance of chromosomes 1A) [48]. These data showed that HSCs survived therapy and were present in the relapse samples, suggesting that they should be targeted therapeutically to treat the disease. Thus, both studies show that SCS methods can provide powerful tools for tracing cell lineages to identify precursor subpopulations and understand how cancer cell lineages relate to normal hematopoietic lineages.

## Measuring mutation rates in single cells

Another major question in cancer biology is whether cancer cells have an increased mutation rate relative to normal cells. The mutator phenotype hypothesis [49] has been posited to be a driving force in tumor progression. The first studies published several decades ago proposed that an increased mutation rate occurred through mutations in DNA polymerases [50], but more recently this model has been extended to include mutations in DNA repair pathways and other genes [49]. Although it is clear from the pan-cancer and The Cancer Genome Atlas (TCGA) studies [51,52] that most human cancers have elevated mutation frequencies (total number of mutations detected at the time of sequencing), it remains unclear whether they have increased mutation rates (more mutations generated after each cell division) or simply more cell divisions at a low mutation rate. The mutation rate of a normal cell has been estimated to be approximately 10^-10^ errors per cell division [53–56], which would generate about one nucleotide error per cell division. The main challenge to obtaining accurate estimates of mutation rates in human tumors is that the number of cell divisions is often difficult to measure. Most tumors do not grow exponentially but reach a plateau phase, in which the number of cell births is equivalent to the number of cell deaths. Human tumors can remain in this equilibrium state for many years, expanding the total size of the tumor at a very slow rate, or not at all.

Bulk-sequencing studies have estimated that the mutation rate across many human cancers is, on average, 210-fold higher than normal cells [57,58]. However, SCS methods can provide far more accurate measures of mutation rates by comparing changes in mutation frequencies from cell to cell. In one study, MALBAC was used to investigate the mutation rate of a human colon cancer cell line [31]. In these experiments, a single cell was subcloned and allowed to expand for 20 cell divisions, after which single-cell whole-genome sequencing was performed. From these data, a mutation rate of 2.5 nucleotide errors per cell division was estimated. As mentioned earlier, NUC-SEQ has been used to investigate the mutation rates of an ER-positive breast cancer and a TNBC from a patient by whole-genome and exome SCS, which showed that the ER-positive breast tumor did not have an increased mutation rate relative to that of normal cells, whereas the TNBC showed a 13.3X increase (eight mutations per cell division) [37]. These mutation rates are substantially lower than previous estimates (210X) made in bulk tissue samples [57,58] but still suggest the existence of an increased mutation rate. However, one caveat is that the SCS studies have only focused on a few patients and single cell lines, and more work is needed to understand the range and extent of mutation rates in human cancers.

## Tracing metastatic dissemination with single circulating tumor cells

CTCs shed from the primary tumor and intravasate into the blood, where they travel to distant organ sites to seed metastatic tumors [59]. Important questions exist regarding the timing of when CTCs disseminate (early or late) [60] and whether they travel unidirectionally or bidirectionally (back and forth, so called self-seeding) between the primary and metastatic tumor sites [61,62]. Another question is whether the metastatic clones are minor subpopulations in the primary tumor that acquire specific genetic mutations that confer metastatic potential or, alternatively, are seeded by the major populations through random shedding into the blood due to leaky angiogenesis in tumors. These questions can be addressed by using single-cell sequencing methods to trace metastatic lineages while utilizing mutations as stable markers of evolution. One of the first pioneering studies in breast cancer showed that CTCs can be enumerated by the presence of the epithelial markers EpCAM and absence of the CD45 immune surface receptors by using the CellSearch system [12]. Data from this study showed that counting five or more CTCs in 7.5 ml of blood has prognostic value in predicting poor five-year survival in patients with metastatic breast cancer. Following this study, enumeration was shown to have prognostic value in predicting survival in many other human cancers [13,63]. However, CTCs are extremely difficult to isolate from the blood as they occur at extremely low frequencies (one in a million mononuclear cells). Consequently, only a few (1 to 50) CTCs can typically be isolated from a 7.5 ml blood draw, which has made the genomic analysis of CTCs very challenging. Hence, the genetic relationships of CTCs to primary and metastatic tumors, and their genomic diversity, remain largely unknown.

The development of SCS methods has enabled researchers to obtain the first genome-wide datasets on CTCs, which is beginning to improve our understanding of their genomic relationship to primary and metastatic tumors. One of the first studies to focus on single-cell transcriptomes used the MagSweeper (Illumina) to isolate CTCs and a microfluidics platform (Fluidigm) to perform multiplexed quantitative PCR (qPCR) on 87 cancer genes in breast cancer cell lines and blood samples from patients [18]. These data showed that single CTC transcriptional profiles of breast cancer samples taken from patients had different expression levels from the breast cancer cell lines, questioning the overall value of using breast cancer cell lines to evaluate the effectiveness of new therapies. Another recent study used the CellSearch system to isolate 37 single CTCs from six patients with metastatic colon cancer for copy-number profiling and targeted NGS using a panel of 68 cancer genes [64]. The data showed that many of the CTC copy-number profiles were similar to those of the primary and metastatic tumor cells, and that point mutations in *APC* (encoding adenomatous polyposis coli protein), *KRAS*, *PIK3CA* (phosphatidylinositol 4,5-bisphosphate 3-kinase catalytic subunit alpha isoform) and *TP53* (cellular tumor antigen p53) in the primary tumors were also present in the single CTCs, suggesting that CTCs will have clinical utility for non-invasive monitoring.

The initial CTC studies were restricted to gene panels and specific transcripts, whereas two recent studies in prostate cancer [65] and lung adenocarcinoma [66] have applied whole-exome sequencing of single CTCs. In the lung cancer study, the exomes of 24 single CTCs, as well as cells from the matched primary and metastatic tumors, were sequenced from four patients using MALBAC [66]. The copy-number profiles of the single CTCs were highly similar and shared most of the same CNAs as the primary and metastatic tumor cells. By contrast, the exome data on point mutations showed extensive variation from cell to cell. This variation might be due to technical errors or real biological heterogeneity; the authors were not able to distinguish between these two possibilities owing to the high FP and ADO error rate of MALBAC. Interestingly, the authors identified a number of CTC-specific mutations that showed no evidence of existing in the primary or metastatic tumors. These mutations are intriguing if they are real biological variants as they would suggest that CTCs continue to evolve new mutations in the circulatory system.

In the prostate cancer study, the authors used a pooling strategy to detect mutations in CTCs to overcome the poor coverage and high ADO rate of single-cell exome sequencing data [65]. Nineteen single CTCs and multiple spatial regions of the primary prostate tumor and the bone metastases were sequenced from a patient with metastatic prostate cancer. To compensate for the low physical coverage and random FP errors that occur in individual CTCs, the authors pooled the single-CTC data together and detected mutations that occur in multiple cells. They found that 51% of the mutations that occurred in the primary and metastatic sites could be detected in the CTCs, and there were also a large number of CTC-specific mutations. Similar to the lung cancer study described above, the CTC-specific mutations were not validated, and thus it remains unclear whether they are technical errors. In summary, these initial studies are very encouraging as they show that a large number of mutations in the primary and metastatic tumors can be detected in CTCs, suggesting that they will have important clinical applications for non-invasive monitoring. This will be discussed further below in the section on clinical applications.

## Transcriptional diversity of single cancer cells

Single-cell transcriptome profiling has begun to unravel the complex admixture of transcriptional profiles that are present in solid tumors and hematopoietic cancers. Initial studies used multiplexed single-cell RT-qPCR to measure the expression levels of hundreds of transcripts in single tumor cells in parallel. In colon cancer, these methods showed that single colon tumor cells have distinct subpopulations of transcriptional profiles that match different cell types in normal epithelial colon tissues [67]. These data identified several transcripts with prognostic value in predicting patient survival. More recently, the field has moved from highly multiplexed qPCR platforms to single-cell RNA-seq, which can profile the entire transcriptome of each individual cancer cell. In a technical study using colon cancer cell lines, it was shown that single-cell multiplexed RT-qPCR could quantify similar expression levels to single-cell RNA-seq, paving the way for future studies [68]. Recently, single-cell RNA-seq was used to study transcriptional diversity in glioblastomas by sequencing 430 cells from five patients [69]. In seminal work leading up to this study, it was shown that glioblastoma patients could be classified into four expression subtypes: classical, neural, proneural and mesenchymal [70]. Single-cell sequencing further showed that, while patients could be classified into these subtypes, many individual tumor cells expressed different subtypes (within the same patient). In contrast to the prevailing paradigm, these data also showed that single cells expressed a broad range of intermediate transcriptional states - from stem cell-like to differentiated - rather than belonging to one distinct group. Future applications of single cell RNA-seq in other cancer types are likely to reveal the importance of stem-like cells and cancer stem cells in tumor progression, and might also provide insight into the cell-of-origin in human cancers.

## Extensive biological diversity or extensive technical errors?

A pervasive problem in SCS studies is that there is often no orthogonal validation performed on the variable mutations or transcriptional changes that are detected in single cells. Validation of SCS results is crucially important owing to the high number of technical errors (FP, FN and ADO) that emerge during WGA or WTA. Alarmingly, these errors are often interpreted as real biological variations at the DNA or RNA level. Some studies have attempted to ‘validate’ single-cell mutations by sequencing the same DNA that has already been WGA amplified. This is by no means an adequate approach for validating mutations as it only eliminates sequencing artifacts and not the most prevalent type of technical errors that arise during the initial rounds of WGA. To perform orthogonal validation, it is necessary to first identify the specific transcripts or mutations that show heterogeneity in a population of cells and validate their variability using an alternative approach.

For RNA experiments, orthogonal validation can be achieved by performing single-cell qPCR on a set of targeted probes or in tissue sections using RNA-FISH. To validate CNAs, FISH probes designed to target specific amplifications or deletions can be used. By hybridizing these probes to tissue sections, it is possible to detect CNAs in thousands of single cells *in situ* with knowledge of their spatial information. For mutations in DNA detected by single-cell exome or genome sequencing, a targeted custom-capture platform can be used to perform ultra-deep sequencing of the cellular DNA from the bulk tumor. However, sequencing technologies have high error rates (approximately 0.1 to 1% for Illumina), which severely limit the accurate detection of mutations that occur at a frequency below 10% in the population. To overcome this limitation, it is necessary to use single-molecule barcoding methods such as duplex sequencing [42] or Safe-Seq [71], which reduce the sequencing error rate from 10^-2^ to 10^-10^. Briefly, these methods add 12 to 24 bp random tags to each molecule in a pool of fragmented DNA and are expanded by PCR to generate 10 to 20 duplicates of each tag. Sequencing errors accumulate randomly in the DNA sequences of the duplicate molecules, and, after sequencing, read groups with common tags are identified. From each group of reads with a common tag, a consensus sequence is calculated that eliminates random errors that accumulated during sequencing, resulting in single-molecule information. Recently, this approach has been used to validate subclonal mutations detected by single-cell exome sequencing in breast tumors [37]. The major advantage of duplex sequencing is that it not only validates subclonal mutations but also provides accurate measures of the mutation frequencies in the bulk tumor cell population by profiling the genotypes of thousands of cells. In summary, owing to the high technical error rates that are inherent in SCS methods, orthogonal validation is of paramount importance. Without validation, many SCS studies are likely to falsely report extensive ‘biological variation’, when in fact they are merely observing extensive ‘technical errors’.

## Clinical applications of single-cell sequencing

SCS methods are expected to have important clinical applications in cancer management within the next five years. These applications include non-invasive monitoring, measuring intratumor heterogeneity, analyzing scarce clinical samples, early detection and guiding targeted therapy towards the malignant tumor cells.

Non-invasive monitoring of CTCs in the blood holds great promise for eliminating the inherent risks that are associated with taking invasive core biopsy samples directly from organ sites (such as infection, internal bleeding and even death). Some of the first SCS studies of CTCs have already shown that a large fraction of the mutations (>50%) detected at the primary and metastatic tumor site can be identified in the CTCs [65,66]. Using CTCs, it is possible to collect and analyze blood samples at multiple time-points during the course of the disease and during treatment. This will enable the oncologist to make rapid changes in therapeutic strategies in response to new mutations emerging during clonal evolution. In addition to monitoring CTCs, short (100 to 200 bp) DNA fragments in the blood plasma called circulating-tumor DNA (ctDNA) can be analyzed by NGS methods [72,73]. To date, however, there have not been direct comparisons of CTCs and ctDNA to determine their detection efficiencies and coverage performance for non-invasive monitoring in patients.

SCS can also be used to measure the extent of intratumor genomic heterogeneity in patients by randomly sampling and sequencing multiple single cells and comparing their mutational profiles to calculate a diversity index. These diversity indexes might correlate with clinical parameters and have prognostic value in predicting response to chemotherapy and survival in patients [74,75]. A tumor with a high diversity index is expected to become resistant to chemotherapy, because it is more likely than a homogenous tumor mass to harbor pre-existing resistance mutations.

Obtaining genomic information from scarce clinical samples using NGS analysis is another important clinical application of SCS. In clinical samples, such as fine-needle aspirates, core biopsy samples, urine, prostate fluid, sperm, feces, lymphatic fluids and blood, the number of tumor cells is often severely limited, but still sufficient for SCS methods. Early detection of cancer could also be improved by using SCS and could be applied to any of the aforementioned clinical samples. In the not-too-distant future, we can imagine a world in which a healthy individual will visit a general practitioner once a year to have their blood drawn. The blood would be processed to identify any CTCs, and the DNA would be sequenced to identify potential driver mutations. The spectrum and combination of mutations in the CTCs or transcriptional profiles could indicate the original organ site from which the CTC had disseminated. The doctors could then follow up with imaging and other biomarkers to identify the tumor at the earliest stages of growth for surgical removal or therapeutic intervention.

A final application of SCS in the clinic is to reconstruct phylogenetic trees and cell lineages to help guide therapeutic targeting. Ideally, oncologists would target mutations that are present in all of the single tumor cells in order to fully eradicate the tumor mass. This would involve targeting the ‘trunk’ or founder mutations in the phylogenetic trees, which are inherited by all subsequent tumor cells. Alternatively, different therapeutic strategies could be devised to target each of the major tumor subpopulations individually.

## Conclusions and future directions

The initial studies on SCS in cancer have shown great promise in improving our understanding of this complex disease and have begun to answer the fundamental questions posed in this review. Although most of these studies have focused on delineating clonal evolution and diversity in primary tumors [30,34,36,37,45,46], the field has begun to shift towards studying CTCs and their role in metastatic dissemination [64–66]. These experiments are likely to provide new insight into understanding the general models of metastasis that have been proposed in human cancers, including early dissemination, late dissemination/parallel evolution and self-seeding or bidirectional trafficking [60,61]. SCS tools are highly advantageous for lineage-tracing studies as mutations in single cells provide stable markers of evolution. One question that has become addressed by the initial single-cell sequencing studies in primary tumors [30,34,36,37,45,46] concerns whether most human tumors originate from a single somatic cell in the normal tissue (not multiple cells). This is supported by a common set of founder mutations that are shared between all single cells in each patient, suggesting an origin from a common ancestor. The initial data comparing CTCs with primary and metastatic tumors [64–66] have already indicated that a large number of similar mutations can be detected (>50%), suggesting a direct genetic lineage. These data hold great promise for clinical applications for non-invasive monitoring.

In the near future, we expect that SCS will be applied to study other areas of cancer research, including the development of early-stage cancers and the evolution of chemoresistance. SCS can be used to study the initial transformation events and the process of invasion, whereby single tumor cells escape the *in situ* regions and invade the surrounding regions. SCS methods also hold great promise for elucidating the role of clonal diversity in response to chemotherapy [75–77], where it is expected that more clonally diverse tumors will be more likely to harbor resistant clones and thus be more likely to evolve resistance. However, major questions exist regarding whether chemoresistant clones pre-exist as rare cells in tumor populations, or whether resistance mutations are acquired spontaneously in response to being challenged by chemotherapeutic agents. While this question has been studied for decades in bacterial cell populations [78], it remains poorly addressed in most human cancers. Furthermore, while no SCS studies have yet investigated cancer stem cells, SCS methods are likely to provide great insights into our understanding of these rare tumor cells, by revealing their genetic and transcriptomic relationship to the major populations of differentiated tumor cells [11,79,80].

Another growing area of cancer research is trying to understand why clonal diversity exists in human cancers. Most studies on clonal diversity to date have been observational, reporting simply that genetic diversity exists in many tumors. Darwinian evolution, in a growth environment with limited resources, would predict that a dominant clone with driver mutations would outcompete the other subpopulations, resulting in a monoclonal population of tumor cells. However, this is not the case in many human cancers, suggesting that clones might cooperate to drive tumor growth through symbiotic relationships [43,44]. One of the first studies examining clonal cooperation was recently published in which *Wnt* signaling in a mouse model of breast cancer was shown to be required for tumor clones to cooperate and drive tumor growth [81]. In future studies of clonal interactions, it will be important to confirm these data back in human tumor samples by using SCS methods to show that the data are physiologically relevant.

Over the next few years, we also expect to see many technological innovations in SCS. While high-coverage (>90%) performance has largely been achieved [30,31,37], current technologies should now focus on mitigating the ADO and FP error rates. In the near future, it might be possible to perform both genome and transcriptome sequencing on the same single cancer cell. This will be highly advantageous as point mutations detected at both the RNA and DNA level can be distinguished from random technical errors with high confidence when they match in both datasets. Furthermore, these data would provide great insight into molecular mechanisms, such as RNA editing and monoallelic expression in human cancer cells.

While much progress has been made in single-cell genome and transcriptome sequencing methods, epigenomic profiling methods have lagged far behind. This is partly due to the fact that most epigenomic sequencing methods (bisulfide sequencing, methylation-specific enzymes) require that a pool of DNA is split into two separate fractions for treatment, which cannot easily be accomplished in a single cell. Another challenge is that epigenetic modifications (for example, cytosine methylation) cannot be amplified as polymerases do not retain these DNA modifications after synthesizing new strands.

Finally, the use of SCS remains out of reach for many research and clinical laboratories because of the high cost and lack of analytical expertise. The cost of SCS is prohibitive for many laboratories as the current price of sequencing the genome or exome of a single cell is equivalent to sequencing a whole human genome (approximately $5000) or exome (approximately $500). However, these costs are directly related to the cost of NGS technologies and should continue to plummet thanks to the fierce industrial competition that fuels technological innovation. In addition, most studies to date use analytical tools such as in-house scripts and processing pipelines that are not easy to reproduce without the necessary infrastructure and bioinformatics expertise. SCS data still suffer from a large number of technical errors and therefore require more extensive post-processing to identify high-confidence mutations. To date, only two methods have been published for analyzing SCS data, including a method to calculate copy-number profiles by density sampling integer estimation [11] and a method to calculate copy-number information from non-uniform MDA sequencing data [82], and these are great resources for the community. More work is still needed to develop computational methods and statistical tools for detecting point mutations, indels and structural variants in single-cell data.

In summary, SCS methods provide a powerful new approach to study the diversity and evolution of single cancer cells. While further technical improvements are still required, the initial application of these tools to study cancer is highly encouraging and has already provided great insight into this complex disease. In the near future, SCS will begin to be applied to the clinic in early detection, prognostics, diagnostics and therapeutic targeting and thereby will have a direct impact on reducing morbidity in many human cancer patients.

## Abbreviations

ADO: Allelic dropout
AML: Acute myeloid leukemia
APC: Encoding adenomatous polyposis coli protein
BGI: Beijing genome institute
CNAs: Copy-number aberrations
CTCs: Circulating tumor cells
ctDNA: Circulating-tumor DNA
DOP-PCR: Degenerative-oligonucleotide-PCR
DTCs: Disseminated tumor cells
FACS: Flow-assisted cell sorting
FFPE: Formalin-fixed paraffin-embedded
FISH: *in situ* hybridization
FLT3-ITD: Receptor-type tyrosine protein kinase internal tandem duplications
FN: False-negative
FP: False-positive
HER2: Receptor tyrosine-protein kinase erbB-2
HSCs: Hematopoietic stem cells
KRAS: Kirsten rat sarcoma viral oncogene homolog
LCM: Laser-capture microdissection
MALBAC: Multiple annealing and looping-based amplification cycles
MDA: Multiple-displacement amplification
MDS: Myelodysplastic syndrome
NGS: Next-generation sequencing
NPM1: Encoding nucleophosmin
PIK3CA: Phosphatidylinositol 4,5-bisphosphate 3-kinase catalytic subunit alpha isoform
qPCR: Quantitative PCR
SCS: Single-cell sequencing
SKY: Spectral karyotyping
SMC1A: Structural maintenance of chromosomes 1A
SNS: Single-nucleus sequencing
TCGA: The cancer genome atlas
TET2: Encoding methylcytosine dioxygenase
TNBC: Triple-negative breast cancer
TP53: Cellular tumor antigen p53
WGA: Whole-genome amplification
WTA: Whole-transcriptome amplification

## References

[1] Brown TM, Fee E (2006). Rudolf Carl Virchow: medical scientist, social reformer, role model. Am J Public Health.

[2] Teixeira MR, Pandis N, Bardi G, Andersen JA, Mitelman F, Heim S (1995). Clonal heterogeneity in breast cancer: karyotypic comparisons of multiple intra- and extra-tumorous samples from 3 patients. Int J Cancer.

[3] Farabegoli F, Santini D, Ceccarelli C, Taffurelli M, Marrano D, Baldini N (2001). Clone heterogeneity in diploid and aneuploid breast carcinomas as detected by FISH. Cytometry.

[4] Fiegl M, Tueni C, Schenk T, Jakesz R, Gnant M, Reiner A, Rudas M, Pirc-Danoewinata H, Marosi C, Huber H, Drach J (1995). Interphase cytogenetics reveals a high incidence of aneuploidy and intra-tumour heterogeneity in breast cancer. Br J Cancer.

[5] Nik-Zainal S, Van Loo P, Wedge DC, Alexandrov LB, Greenman CD, Lau KW, Raine K, Jones D, Marshall J, Ramakrishna M, Shlien A, Cooke SL, Hinton J, Menzies A, Stebbings LA, Leroy C, Jia M, Rance R, Mudie LJ, Gamble SJ, Stephens PJ, McLaren S, Tarpey PS, Papaemmanuil E, Davies HR, Varela I, McBride DJ, Bignell GR, Leung K, Butler AP (2012). The life history of 21 breast cancers. Cell.

[6] Van Loo P, Campbell PJ (2012). ABSOLUTE cancer genomics. Nat Biotechnol.

[7] Shah SP, Roth A, Goya R, Oloumi A, Ha G, Zhao Y, Turashvili G, Ding J, Tse K, Haffari G, Bashashati A, Prentice LM, Khattra J, Burleigh A, Yap D, Bernard V, McPherson A, Shumansky K, Crisan A, Giuliany R, Heravi-Moussavi A, Rosner J, Lai D, Birol I, Varhol R, Tam A, Dhalla N, Zeng T, Ma K, Chan SK (2012). The clonal and mutational evolution spectrum of primary triple-negative breast cancers. Nature.

[8] Gerlinger M, Rowan AJ, Horswell S, Larkin J, Endesfelder D, Gronroos E, Martinez P, Matthews N, Stewart A, Tarpey P, Varela I, Phillimore B, Begum S, McDonald NQ, Butler A, Jones D, Raine K, Latimer C, Santos CR, Nohadani M, Eklund AC, Spencer-Dene B, Clark G, Pickering L, Stamp G, Gore M, Szallasi Z, Downward J, Futreal PA, Swanton C (2012). Intratumor heterogeneity and branched evolution revealed by multiregion sequencing. N Engl J Med.

[9] Adams JM, Strasser A (2008). Is tumor growth sustained by rare cancer stem cells or dominant clones?. Cancer Res.

[10] Shackleton M, Quintana E, Fearon ER, Morrison SJ (2009). Heterogeneity in cancer: cancer stem cells versus clonal evolution. Cell.

[11] Tomasson MH (2009). Cancer stem cells: a guide for skeptics. J Cell Biochem.

[12] Cristofanilli M, Budd GT, Ellis MJ, Stopeck A, Matera J, Miller MC, Reuben JM, Doyle GV, Allard WJ, Terstappen LW, Hayes DF (2004). Circulating tumor cells, disease progression, and survival in metastatic breast cancer. N Engl J Med.

[13] Allard WJ, Matera J, Miller MC, Repollet M, Connelly MC, Rao C, Tibbe AG, Uhr JW, Terstappen LW (2004). Tumor cells circulate in the peripheral blood of all major carcinomas but not in healthy subjects or patients with nonmalignant diseases. Clin Cancer Res.

[14] Yu M, Stott S, Toner M, Maheswaran S, Haber DA (2011). Circulating tumor cells: approaches to isolation and characterization. J Cell Biol.

[15] Altomare L, Borgatti M, Medoro G, Manaresi N, Tartagni M, Guerrieri R, Gambari R (2003). Levitation and movement of human tumor cells using a printed circuit board device based on software-controlled dielectrophoresis. Biotechnol Bioeng.

[16] Choi JH, Ogunniyi AO, Du M, Du M, Kretschmann M, Eberhardt J, Love JC (2010). Development and optimization of a process for automated recovery of single cells identified by microengraving. Biotechnol Prog.

[17] Nagrath S, Sequist LV, Maheswaran S, Bell DW, Irimia D, Ulkus L, Smith MR, Kwak EL, Digumarthy S, Muzikansky A, Ryan P, Balis UJ, Tompkins RG, Haber DA, Toner M (2007). Isolation of rare circulating tumour cells in cancer patients by microchip technology. Nature.

[18] Powell AA, Talasaz AH, Zhang H, Coram MA, Reddy A, Deng G, Telli ML, Advani RH, Carlson RW, Mollick JA, Sheth S, Kurian AW, Ford JM, Stockdale FE, Quake SR, Pease RF, Mindrinos MN, Bhanot G, Dairkee SH, Davis RW, Jeffrey SS (2012). Single cell profiling of circulating tumor cells: transcriptional heterogeneity and diversity from breast cancer cell lines. PLoS One.

[19] Adams DL, Martin SS, Alpaugh RK, Charpentier M, Tsai S, Bergan RC, Ogden IM, Catalona W, Chumsri S, Tang CM, Cristofanilli M (2014). Circulating giant macrophages as a potential biomarker of solid tumors. Proc Natl Acad Sci U S A.

[20] Hashimshony T, Wagner F, Sher N, Yanai I (2012). CEL-Seq: single-cell RNA-Seq by multiplexed linear amplification. Cell Rep.

[21] Tang F, Barbacioru C, Wang Y, Nordman E, Lee C, Xu N, Wang X, Bodeau J, Tuch BB, Siddiqui A, Lao K, Surani MA (2009). mRNA-Seq whole-transcriptome analysis of a single cell. Nat Methods.

[22] Ramskold D, Luo S, Wang YC, Li R, Deng Q, Faridani OR, Daniels GA, Khrebtukova I, Loring JF, Laurent LC, Schroth GP, Sandberg R (2012). Full-length mRNA-Seq from single-cell levels of RNA and individual circulating tumor cells. Nat Biotechnol.

[23] Islam S, Kjallquist U, Moliner A, Zajac P, Fan JB, Lonnerberg P, Linnarsson S (2011). Characterization of the single-cell transcriptional landscape by highly multiplex RNA-seq. Genome Res.

[24] Sasagawa Y, Nikaido I, Hayashi T, Danno H, Uno KD, Imai T, Ueda HR (2013). Quartz-Seq: a highly reproducible and sensitive single-cell RNA sequencing method, reveals non-genetic gene-expression heterogeneity. Genome Biol.

[25] Pan X, Durrett RE, Zhu H, Tanaka Y, Li Y, Zi X, Marjani SL, Euskirchen G, Ma C, Lamotte RH, Park IH, Snyder MP, Mason CE, Weissman SM (2013). Two methods for full-length RNA sequencing for low quantities of cells and single cells. Proc Natl Acad Sci U S A.

[26] Sandberg R (2014). Entering the era of single-cell transcriptomics in biology and medicine. Nat Methods.

[27] Tang F, Lao K, Surani MA (2011). Development and applications of single-cell transcriptome analysis. Nat Methods.

[28] Lasken RS (2007). Single-cell genomic sequencing using Multiple Displacement Amplification. Curr Opin Microbiol.

[29] Dean FB, Hosono S, Fang L, Wu X, Faruqi AF, Bray-Ward P, Sun Z, Zong Q, Du Y, Du J, Driscoll M, Song W, Kingsmore SF, Egholm M, Lasken RS (2002). Comprehensive human genome amplification using multiple displacement amplification. Proc Natl Acad Sci U S A.

[30] Hou Y, Song L, Zhu P, Zhang B, Tao Y, Xu X, Li F, Wu K, Liang J, Shao D, Wu H, Ye X, Ye C, Wu R, Jian M, Chen Y, Xie W, Zhang R, Chen L, Liu X, Yao X, Zheng H, Yu C, Li Q, Gong Z, Mao M, Yang X, Yang L, Li J, Wang W (2012). Single-cell exome sequencing and monoclonal evolution of a JAK2-negative myeloproliferative neoplasm. Cell.

[31] Zong C, Lu S, Chapman AR, Xie XS (2012). Genome-wide detection of single-nucleotide and copy-number variations of a single human cell. Science.

[32] Fiegler H, Geigl JB, Langer S, Rigler D, Porter K, Unger K, Carter NP, Speicher MR (2007). High resolution array-CGH analysis of single cells. Nucleic Acids Res.

[33] Talseth-Palmer BA, Bowden NA, Hill A, Meldrum C, Scott RJ (2008). Whole genome amplification and its impact on CGH array profiles. BMC Res Notes.

[34] Navin N, Kendall J, Troge J, Andrews P, Rodgers L, McIndoo J, Cook K, Stepansky A, Levy D, Esposito D, Muthuswamy L, Krasnitz A, McCombie WR, Hicks J, Wigler M (2011). Tumour evolution inferred by single-cell sequencing. Nature.

[35] Baslan T, Kendall J, Rodgers L, Cox H, Riggs M, Stepansky A, Troge J, Ravi K, Esposito D, Lakshmi B, Wigler M, Navin N, Hicks J (2012). Genome-wide copy number analysis of single cells. Nat Protoc.

[36] Xu X, Hou Y, Yin X, Bao L, Tang A, Song L, Li F, Tsang S, Wu K, Wu H, He W, Zeng L, Xing M, Wu R, Jiang H, Liu X, Cao D, Guo G, Hu X, Gui Y, Li Z, Xie W, Sun X, Shi M, Cai Z, Wang B, Zhong M, Li J, Lu Z, Gu N (2012). Single-cell exome sequencing reveals single-nucleotide mutation characteristics of a kidney tumor. Cell.

[37] Wang Y, Waters J, Leung ML, Unruh A, Roh W, Shi X, Chen K, Scheet P, Vattathil S, Liang H, Multani A, Zhang H, Zhao R, Michor F, Meric-Bernstam F, Navin NE (2014). Clonal evolution in breast cancer revealed by single nucleus genome sequencing. Nature.

[38] Lasken RS (2013). Single-cell sequencing in its prime. Nat Biotechnol.

[39] Van Loo P, Voet T (2014). Single cell analysis of cancer genomes. Curr Opin Genet Dev.

[40] Navin NE, Hicks J (2010). Tracing the tumor lineage. Mol Oncol.

[41] Fearon ER, Vogelstein B (1990). A genetic model for colorectal tumorigenesis. Cell.

[42] Schmitt MW, Kennedy SR, Salk JJ, Fox EJ, Hiatt JB, Loeb LA (2012). Detection of ultra-rare mutations by next-generation sequencing. Proc Natl Acad Sci U S A.

[43] Merlo LMF, Pepper JW, Reid BJ, Maley CC (2006). Cancer as an evolutionary and ecological process. Nat Rev Cancer.

[44] Greaves M, Maley CC (2012). Clonal evolution in cancer. Nature.

[45] Li Y, Xu X, Song L, Hou Y, Li Z, Tsang S, Li F, Im KM, Wu K, Wu H, Ye X, Li G, Wang L, Zhang B, Liang J, Xie W, Wu R, Jiang H, Liu X, Yu C, Zheng H, Jian M, Nie L, Wan L, Shi M, Sun X, Tang A, Guo G, Gui Y, Cai Z (2012). Single-cell sequencing analysis characterizes common and cell-lineage-specific mutations in a muscle-invasive bladder cancer. Gigascience.

[46] Yu C, Yu J, Yao X, Wu WK, Lu Y, Tang S, Li X, Bao L, Li X, Hou Y, Wu R, Jian M, Chen R, Zhang F, Xu L, Fan F, He J, Liang Q, Wang H, Hu X, He M, Zhang X, Zheng H, Li Q, Wu H, Chen Y, Yang X, Zhu S, Xu X, Yang H (2014). Discovery of biclonal origin and a novel oncogene SLC12A5 in colon cancer by single-cell sequencing. Cell Res.

[47] Hughes AE, Magrini V, Demeter R, Miller CA, Fulton R, Fulton LL, Eades WC, Elliott K, Heath S, Westervelt P, Ding L, Conrad DF, White BS, Shao J, Link DC, DiPersio JF, Mardis ER, Wilson RK, Ley TJ, Walter MJ, Graubert TA (2014). Clonal architecture of secondary acute myeloid leukemia defined by single-cell sequencing. PLoS Genet.

[48] Jan M, Snyder TM, Corces-Zimmerman MR, Vyas P, Weissman IL, Quake SR, Majeti R: **Clonal evolution of preleukemic hematopoietic stem cells precedes human acute myeloid leukemia.** *Sci Transl Med* 2012, **4**:ᅟ. 149ra118. 10.1126/scitranslmed.3004315 PMC4045621 22932223

[49] Loeb LA (2011). Human cancers express mutator phenotypes: origin, consequences and targeting. Nat Rev Cancer.

[50] Loeb LA, Springgate CF, Battula N (1974). Errors in DNA replication as a basis of malignant changes. Cancer Res.

[51] Kandoth C, McLellan MD, Vandin F, Ye K, Niu B, Lu C, Xie M, Zhang Q, McMichael JF, Wyczalkowski MA, Leiserson MD, Miller CA, Welch JS, Walter MJ, Wendl MC, Ley TJ, Wilson RK, Raphael BJ, Ding L (2013). Mutational landscape and significance across 12 major cancer types. Nature.

[52] Alexandrov LB, Nik-Zainal S, Wedge DC, Aparicio SA, Behjati S, Biankin AV, Bignell GR, Bolli N, Borg A, Borresen-Dale AL, Boyault S, Burkhardt B, Butler AP, Caldas C, Davies HR, Desmedt C, Eils R, Eyfjord JE, Foekens JA, Greaves M, Hosoda F, Hutter B, Ilicic T, Imbeaud S, Imielinski M, Jager N, Jones DT, Jones D, Knappskog S, Kool M (2013). Signatures of mutational processes in human cancer. Nature.

[53] Lynch M (2010). Evolution of the mutation rate. Trends Genet.

[54] Drake JW (1999). The distribution of rates of spontaneous mutation over viruses, prokaryotes, and eukaryotes. Ann N Y Acad Sci.

[55] Nachman MW, Crowell SL (2000). Estimate of the mutation rate per nucleotide in humans. Genetics.

[56] Preston BD, Albertson TM, Herr AJ (2010). DNA replication fidelity and cancer. Semin Cancer Biol.

[57] Bielas JH, Loeb KR, Rubin BP, True LD, Loeb LA (2006). Human cancers express a mutator phenotype. Proc Natl Acad Sci U S A.

[58] Bielas JH, Loeb LA (2005). Mutator phenotype in cancer: timing and perspectives. Environ Mol Mutagen.

[59] Valastyan S, Weinberg RA (2011). Tumor metastasis: molecular insights and evolving paradigms. Cell.

[60] Klein CA (2009). Parallel progression of primary tumours and metastases. Nat Rev Cancer.

[61] Norton L, Massague J (2006). Is cancer a disease of self-seeding?. Nat Med.

[62] Kim MY, Oskarsson T, Acharyya S, Nguyen DX, Zhang XH, Norton L, Massague J (2009). Tumor self-seeding by circulating cancer cells. Cell.

[63] Garcia JA, Rosenberg JE, Weinberg V, Scott J, Frohlich M, Park JW, Small EJ (2007). Evaluation and significance of circulating epithelial cells in patients with hormone-refractory prostate cancer. BJU Int.

[64] Heitzer E, Auer M, Gasch C, Pichler M, Ulz P, Hoffmann EM, Lax S, Waldispuehl-Geigl J, Mauermann O, Lackner C, Hofler G, Eisner F, Sill H, Samonigg H, Pantel K, Riethdorf S, Bauernhofer T, Geigl JB, Speicher MR (2013). Complex tumor genomes inferred from single circulating tumor cells by array-CGH and next-generation sequencing. Cancer Res.

[65] Lohr JG, Adalsteinsson VA, Cibulskis K, Choudhury AD, Rosenberg M, Cruz-Gordillo P, Francis JM, Zhang CZ, Shalek AK, Satija R, Trombetta JJ, Lu D, Tallapragada N, Tahirova N, Kim S, Blumenstiel B, Sougnez C, Lowe A, Wong B, Auclair D, Van Allen EM, Nakabayashi M, Lis RT, Lee GS, Li T, Chabot MS, Ly A, Taplin ME, Clancy TE, Loda M (2014). Whole-exome sequencing of circulating tumor cells provides a window into metastatic prostate cancer. Nat Biotechnol.

[66] Ni X, Zhuo M, Su Z, Duan J, Gao Y, Wang Z, Zong C, Bai H, Chapman AR, Zhao J, Xu L, An T, Ma Q, Wang Y, Wu M, Sun Y, Wang S, Li Z, Yang X, Yong J, Su XD, Lu Y, Bai F, Xie XS, Wang J (2013). Reproducible copy number variation patterns among single circulating tumor cells of lung cancer patients. Proc Natl Acad Sci U S A.

[67] Dalerba P, Kalisky T, Sahoo D, Rajendran PS, Rothenberg ME, Leyrat AA, Sim S, Okamoto J, Johnston DM, Qian D, Zabala M, Bueno J, Neff NF, Wang J, Shelton AA, Visser B, Hisamori S, Shimono Y, van de Wetering M, Clevers H, Clarke MF, Quake SR (2011). Single-cell dissection of transcriptional heterogeneity in human colon tumors. Nat Biotechnol.

[68] Wu AR, Neff NF, Kalisky T, Dalerba P, Treutlein B, Rothenberg ME, Mburu FM, Mantalas GL, Sim S, Clarke MF, Quake SR (2014). Quantitative assessment of single-cell RNA-sequencing methods. Nat Methods.

[69] Patel AP, Tirosh I, Trombetta JJ, Shalek AK, Gillespie SM, Wakimoto H, Cahill DP, Nahed BV, Curry WT, Martuza RL, Louis DN, Rozenblatt-Rosen O, Suva ML, Regev A, Bernstein BE (2014). Single-cell RNA-seq highlights intratumoral heterogeneity in primary glioblastoma. Science.

[70] Verhaak RG, Hoadley KA, Purdom E, Wang V, Qi Y, Wilkerson MD, Miller CR, Ding L, Golub T, Mesirov JP, Alexe G, Lawrence M, O'Kelly M, Tamayo P, Weir BA, Gabriel S, Winckler W, Gupta S, Jakkula L, Feiler HS, Hodgson JG, James CD, Sarkaria JN, Brennan C, Kahn A, Spellman PT, Wilson RK, Speed TP, Gray JW, Meyerson M (2010). Integrated genomic analysis identifies clinically relevant subtypes of glioblastoma characterized by abnormalities in PDGFRA, IDH1, EGFR, and NF1. Cancer Cell.

[71] Kinde I, Wu J, Papadopoulos N, Kinzler KW, Vogelstein B (2011). Detection and quantification of rare mutations with massively parallel sequencing. Proc Natl Acad Sci U S A.

[72] Dawson SJ, Tsui DW, Murtaza M, Biggs H, Rueda OM, Chin SF, Dunning MJ, Gale D, Forshew T, Mahler-Araujo B, Rajan S, Humphray S, Becq J, Halsall D, Wallis M, Bentley D, Caldas C, Rosenfeld N (2013). Analysis of circulating tumor DNA to monitor metastatic breast cancer. N Engl J Med.

[73] Forshew T, Murtaza M, Parkinson C, Gale D, Tsui DW, Kaper F, Dawson SJ, Piskorz AM, Jimenez-Linan M, Bentley D, Hadfield J, May AP, Caldas C, Brenton JD, Rosenfeld N: **Noninvasive identification and monitoring of cancer mutations by targeted deep sequencing of plasma DNA.** *Sci Transl Med* 2012, **4**:ᅟ. 136ra168. 10.1126/scitranslmed.3003726 22649089

[74] Burrell RA, McGranahan N, Bartek J, Swanton C (2013). The causes and consequences of genetic heterogeneity in cancer evolution. Nature.

[75] Almendro V, Cheng YK, Randles A, Itzkovitz S, Marusyk A, Ametller E, Gonzalez-Farre X, Munoz M, Russnes HG, Helland A, Rye IH, Borresen-Dale AL, Maruyama R, van Oudenaarden A, Dowsett M, Jones RL, Reis-Filho J, Gascon P, Gonen M, Michor F, Polyak K (2014). Inference of tumor evolution during chemotherapy by computational modeling and in situ analysis of genetic and phenotypic cellular diversity. Cell Rep.

[76] Navin NE (2014). Tumor evolution in response to chemotherapy: phenotype versus genotype. Cell Rep.

[77] Ding L, Ley TJ, Larson DE, Miller CA, Koboldt DC, Welch JS, Ritchey JK, Young MA, Lamprecht T, McLellan MD, McMichael JF, Wallis JW, Lu C, Shen D, Harris CC, Dooling DJ, Fulton RS, Fulton LL, Chen K, Schmidt H, Kalicki-Veizer J, Magrini VJ, Cook L, McGrath SD, Vickery TL, Wendl MC, Heath S, Watson MA, Link DC, Tomasson MH (2012). Clonal evolution in relapsed acute myeloid leukaemia revealed by whole-genome sequencing. Nature.

[78] Luria SE, Delbruck M (1943). Mutations of bacteria from virus sensitivity to virus resistance. Genetics.

[79] Shipitsin M, Polyak K (2008). The cancer stem cell hypothesis: in search of definitions, markers, and relevance. Lab Invest.

[80] Stingl J, Caldas C (2007). Molecular heterogeneity of breast carcinomas and the cancer stem cell hypothesis. Nat Rev Cancer.

[81] Cleary AS, Leonard TL, Gestl SA, Gunther EJ (2014). Tumour cell heterogeneity maintained by cooperating subclones in Wnt-driven mammary cancers. Nature.

[82] Zhang C, Zhang C, Chen S, Yin X, Pan X, Lin G, Tan Y, Tan K, Xu Z, Hu P, Li X, Chen F, Xu X, Li Y, Zhang X, Jiang H, Wang W (2013). A single cell level based method for copy number variation analysis by low coverage massively parallel sequencing. PLoS One.

